# Densification characteristics of chromia/alumina castables by particle size distribution

**DOI:** 10.1186/1556-276X-7-8

**Published:** 2012-01-05

**Authors:** Jingming Zhao, Taesuk Kim, Gichul Kim, Kyuhong Hwang, Dongsik Bae

**Affiliations:** 1 Engineering Research Institute, i-Cube Center, Gyeongsang National University, Jinju, 660-701, South Korea; 2 Changwon National University, Changwon, 641-773, South Korea

**Keywords:** densification, chromia/alumina, castables, IGCC

## Abstract

The quality of the refractories applied on integrated gasification combined cycle should be a key factor that affects both the reliability and the economics of gasifier operation. To enhance the workability of chromia/alumina castables, three types of ultrafine alumina powder were added to improve the workability. Densification behavior of such castables in the presence of ultrafine alumina was assessed through the measurement of parameters like flow value, viscosity, bulk density, apparent porosity, and microstructure evaluation by an SEM study. It's proved that the specific surface area and particle size distribution of ultrafine powders in matrix parts greatly influence the densification behavior of these castables.

## Introduction

The gasification process converts carbonaceous materials such as coal, petroleum coke, and biomass to synthesis gas consisting of H_2 _and CO that can be utilized as a chemical feedstock or for powder generation. The ash from the carbon feedstock is liquefied into slag in the gasification chamber and can corrode, penetrate, and interact with the refractory liner at the elevated temperatures, severely limiting refractory service life and gasifier operation [1]. Reaction can occur between refractory materials and slag oxides of Fe, Si, and/or V or with H_2 _and CO gasification products.

Chromia/alumina castables have been widely used in integrated gasification combined cycle [IGCC]. It provides a number of advantages such as high resistance to slag corrosion and low slag penetration. However, the current generation refractory liners installed in gasifier systems have a short service life [2]. This paper discusses efforts to increase refractory service life through the development of refractory densification. The densification effect was examined by adding ultrafine alumina powder to reduce the amount of water and improve the flow ability of the chromia/alumina refractory castable.

## Experimental procedure

The particle size distribution of the castable was adjusted to a theoretical self-flowing continuous curve based on the Andreassen model [3]. The white fused alumina was used as aggregate, and the distributions were 3 to approximately 5 mm, 1 to approximately 3 mm, and approximately 1 mm with optimized distribution. Chromia and alumina were used as matrix taken at mass ratios of 0, 0.5, and 1.0. The following additions were introduced into the mixture: high grade alumina cement, 3%; ultrafine fumed silica powder, 1%; two types of polycarboxylate ether based on polycarboxylic acid, 0.1%. Three types of ultrafine alumina were added to enhance the flow ability of the matrix powders, and their physical properties are shown in Table 1.

**Table 1:** The properties of active alumina

Property/method	Unit	CTC-20	CTC-30	CTC-40
Na_2_O	%	0.12	0.08	0.08
Fe_2_O_3_	%	0.03	0.02	0.03
MgO	%	0.01	0.04	0.03
SiO_2_	%	0.03	0.03	0.03
CaO	%	0.02	0.02	0.03
Specific surface area/BET	m^2^/g	2.1	3.8	4.8
D50 Cilas	μm	1.9	1.8	1.4
D90 Cilas	μm	5.4	6.0	6.0
Particle size distribution	Peak	Unimodal	Multimodal	Bimodal

The prepared mix was poured into steel molds with dimensions of 150 × 15 × 15 mm and 50 × 50 × 50 mm for bending and compressive strength tests, respectively. After being cured for 1 day in air, the demoded samples were put into a drying oven for 24 h at 110°C. Then, the dried samples were sintered at 1,300°C and 1,600°C, respectively for 3 h with a temperature elevation of 5°C/min.

Physical properties such as apparent porosity, bulk density, and water absorption were measured. CCS was carried out, and the CAS formation was observed by a scanning electron microscope.

## Result and discussion

**Figure 1:**
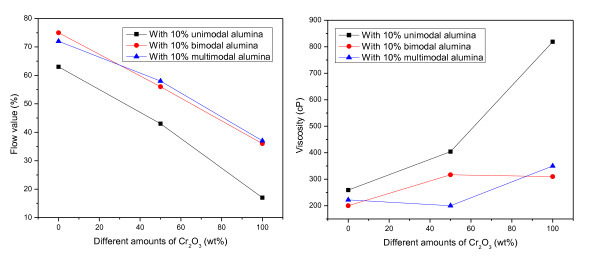
**The effects of the addition of ultrafine active alumina on the workabilities of castables**.

The chromia/alumina castable has an excellent resistance to slag corrosion. To provide the optimum particle packing, the grain composition of fine alumina powders and alumina cement in matrix parts in the castable would be an essential requirement to obtain a castable associating high flow ability with low water content. The flow value and viscosity of the specimens with different ultrafine alumina powders were tested as shown in Figure 1. The viscosity of the matrix powders decreases with the addition of multimodal and bimodal alumina powders. So, more fine particles which can be packed between matrix powders would be a good method for decreasing viscosity, that is, increasing workability.

**Figure 2:**
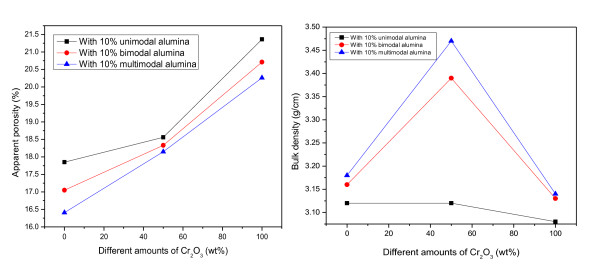
**Effects of adding ultrafine active alumina on the apparent porosities and bulk densities of castables**.

For the batch containing the ultrafine alumina powder additive, bulk density values also remained more or less the same with an increase in firing temperature. However, the multimodal powder was more effective in workability than the unimodal alumina, so the bulk density was greatly increased and apparent porosity was decreased as shown in Figure 2. Reduction of the apparent porosity of the castable should lead to a lower hot slag and metal penetration, reduced spalling, and therefore, increased lining life in steel-making applications.

**Figure 3:**
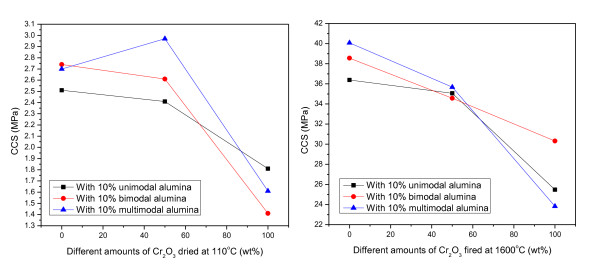
**Effects of adding ultrafine active alumina on the cold crushing strength of the castables**. The castables were subjected with different amounts of Cr_2_O_3 _at temperatures of 110°C and 1,600°C.

Cold crushing strength results are presented as a function of firing temperatures at 110°C and 1,600°C in Figure 3. With the amounts of 0% and 50% Cr_2_O_3_, the castable which contains the multimodal alumina mixture shows the highest values of strength which are related to the higher amount of the cement-hydrated products CAH_10 _and AH_3 _that play the role of bonding. However, further increment in the Cr_2_O_3 _up to 100 wt.% results in a great deterioration in strength due to low workability.

**Figure 4:**
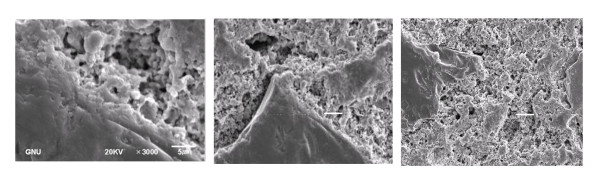
**The variation of microstructures of castables due to the different ultrafine alumina powders in matrix**. (**a**) Unimodal alumina, (**b**) bimodal alumina, and (**c**) multimodal alumina.

From the scanning electron micrographs of the fired samples (Figure 4), it was observed that ultrafine alumina powder additions caused the relative proportion of crystalline phases to increase and the amorphous phases to decrease. The average grain size of the crystalline phases decreased with an increase in ultrafine alumina powder additive. All the formation of the glassy phases and porosity development were found to be less in the batch with ultrafine alumina powder additive, and the microstructure was more uniform. However, densification composition was observed for the multimodal alumina addition in Figure 4.

## Conclusions

To improve the mechanical and chemical properties of the chromia/alumina castable, which is widely used in IGCC application, the ultrafine alumina powder with different particle size distribution which was added as a matrix fine powder was investigated.

1. With the ultrafine alumina addition, the amount of water could be controlled below 5% even in chromia containing alumina castables so that it can fabricate the densified refractory structure.

2. Ultrafine alumina powder added into the chromia/alumina castable can greatly increase the strength of specimens after sintering; the workability can be enhanced so that it can show high densification and high strength after firing.

3. Comparing the three ultrafine alumina powders, multimodal alumina is recommended to get a dense body because of the good particle size distribution in the castable.

4. The bulk density was increased especially at 50% chromia contents so the strength shows high values after firing at a high temperature.

## Competing interests

The authors declare that they have no competing interests.

## Authors' contributions

JZ carried out the full castable studies and participated in modifying and drafting the manuscript. TK participated in the sequence alignment. GK and DB participated in the design of the study and performed the statistical analysis. KH conceived of the study and participated in its design and coordination. All authors read and approved the final manuscript.
